# Inhibition of CD38 by 78c Enhanced NAD^+^ and Alleviated Alveolar Bone Loss in Mice with Experimental Periodontitis

**DOI:** 10.3390/ijms27135829

**Published:** 2026-06-28

**Authors:** Jon Stephen Yarbrough, Subramanya Pandruvada, William D. Hill, Hong Yu

**Affiliations:** 1 Department of Biomedical and Community Health Sciences, College of Dental Medicine, Medical University of South Carolina, Charleston, SC 29425, USA; jsy300@musc.edu (J.S.Y.); pandruv@musc.edu (S.P.); 2 Department of Pathology and Laboratory Medicine, Medical University of South Carolina, Charleston, SC 29425, USA; hillwi@musc.edu

**Keywords:** CD38, NAD^+^, age, cytokine, osteoclastogenesis, periodontitis

## Abstract

Old murine bone marrow-derived monocytes and macrophages (BMMs) display enhanced CD38 protein, a nicotinamide adenine dinucleotide (NAD^+^) glycohydrolase, and reduced NAD^+^ level after infection with oral pathogens compared to young controls. We aimed to determine whether treatment with a CD38-specific inhibitor (78c) in mice with experimental periodontitis could alleviate alveolar bone loss and enhance NAD^+^ levels in tissues compared with vehicle treatment. Twenty young (2-month-old) and twenty old (18-month-old) male C57BL/6J mice with experimental periodontitis were treated with either vehicle or 78c twice daily via intraperitoneal injection for 4 weeks. The liver, spleen, and right maxillary tissues were harvested to analyze NAD^+^ levels. The left maxillary tissues were scanned by micro-CT. Then, the left maxillary tissues were processed for tissue sectioning and stained with hematoxylin and eosin (H&E) and tartrate−resistant acid phosphatase (TRAP). Treatment with 78c significantly enhanced NAD^+^ levels in the liver and spleen of both young and old mice, and significantly increased NAD^+^ in the right maxilla of old mice compared with vehicle treatment. Additionally, treatment with 78c alleviated alveolar bone loss in both young and old mice. Our results support the notion that 78c is a promising therapeutic strategy for treating periodontal disease.

## 1. Introduction

Periodontitis, an inflammatory bone loss disease, is the 6th most prevalent disease, affecting 734 million people worldwide [1]. The incidence of periodontitis among adults in the United States is 47% [2]. With aging, individuals increase both the prevalence and the severity of periodontal disease. Approximately 70% of people over 65 years old in the United States have periodontal disease [3]. This increase in prevalence and severity of periodontal disease in older adults is associated with comorbid systemic diseases, poor physical functioning, inflammatory dysregulation, and limited ability to self-care in older populations [4,5]. One characteristic of periodontitis is that oral bacterial pathogens activate toll-like receptor (TLR) pathways, leading to elevated expression of pro-inflammatory cytokines (such as IL-1β, IL-6, and ΤNF) in periodontal tissues. Another major characteristic of periodontitis is that periodontal inflammation promotes osteoclastogenesis and subsequent alveolar bone loss.

Cluster of Differentiation 38 (CD38) is a type II transmembrane protein ubiquitously expressed in most tissues and cells in mice and humans [6]. CD38 is predominantly expressed in inflammatory cells, including plasma cells, B cells, T cells, natural killer cells, dendritic cells, monocytes, macrophages, and neutrophils [6]. CD38 is a nicotinamide adenine dinucleotide (NAD^+^) glycohydrolase, which degrades NAD^+^ and generates nicotinamide (NAM), ADP-ribose (ADPR), and cyclic ADP-ribose (cADPR) [6,7,8,9]. NAD^+^ can be reduced to NADH by dehydrogenases or can be phosphorylated to NADP^+^ by NAD^+^ kinase [10]. The NAD^+^/NADH couple controls cellular energy generation, glycolysis, and mitochondrial oxidative phosphorylation. In contrast, NADP^+^/NADPH regulates redox homeostasis and supports the biosynthesis of fatty and nucleic acids [10]. Therefore, it is essential to maintain normal NAD^+^ levels to support cellular energy generation, cell metabolism, and redox homeostasis. An increase in CD38 level was observed with aging [11,12], which may be associated with increased aging-related inflammation through a process called inflammaging [13,14]. Increased CD38 during aging leads to further NAD^+^ depletion [15,16,17]. Our previous study [18] demonstrated that old murine bone marrow-derived monocytes and macrophages (BMMs) from 18-month-old C57BL/6J mice showed increased CD38 protein expression and reduced NAD^+^ levels following infection with oral pathogens, such as *Aggregatibacter actinomycetemcomitans* (*Aa*) and *Porphyromonas gingivalis* (*Pg*), compared to young controls. The reduction in NAD^+^ levels in the aging population impacts numerous age-related immune dysfunctions. These include compromised mitochondrial function, buildup of oxidatively damaged molecules within cells, disrupted energy metabolism, waste removal, and stress response, depletion of stem cells, and impaired DNA repair and neuronal calcium signaling [15]. Therefore, the decline of NAD^+^ contributes to the pathogenesis of various aging-associated diseases, including infection, neurodegenerative diseases [15,16,17], cancer [7], and type II diabetes [7,16,19].

Oral bacterial pathogens stimulate TLRs and their downstream signaling pathways [20,21,22,23,24], such as phosphoinositide 3-kinase (PI3K), nuclear factor kappa-B (NF-κB), and mitogen-activated protein kinases (MAPKs) [including extracellular signal-regulated kinases (ERKs), c-Jun N-terminal kinase (JNK), and p38 MAPK] [25,26]. Activation of these protein kinases leads to the production of pro-inflammatory cytokines [such as IL-1β, IL-6, ΤNF-α, and receptor activator of NF-κB ligand (RANKL)], which subsequently cause periodontal tissue damage and alveolar bone loss. Our previous study [27] demonstrated that treatment with the CD38-specific inhibitor (78c) dose-dependently suppressed CD38 mRNA levels, reversed the decline in NAD^+^, and decreased IL-1β, IL-6, and TNF-α levels in murine BMMs infected with oral pathogens (*Aa* or *Pg*) compared with vehicle controls. Mechanistically, we demonstrated that treatment with 78c inhibited PI3K, NF-κB, and MAPKs induced by oral pathogens [27].

Furthermore, our earlier study [27] showed that treatment with 78c suppressed RANKL-induced osteoclastogenesis and bone resorption. Mechanistically, we demonstrated that treatment with 78c suppressed podosome (basic cell adhesion unit) components, including filamentous actin (F-actin), PI3K, Pyk2, Src, integrins, paxillin, and talin. Inhibition of these cellular adhesive components leads to suppression of the adhesion and fusion of monocytes and macrophages (osteoclast precursors), which adhere and fuse to form multinucleated osteoclasts [27]. In this study, we hypothesized that treating mice with experimental periodontitis with the CD38-specific inhibitor (78c) could alleviate alveolar bone loss and increase NAD^+^ levels in tissues.

## 2. Results

### 2.1. Old Mice with Experimental Periodontitis Experienced a Significant Loss in Body Weight Compared to Young Mouse Controls

**Figure 1:**
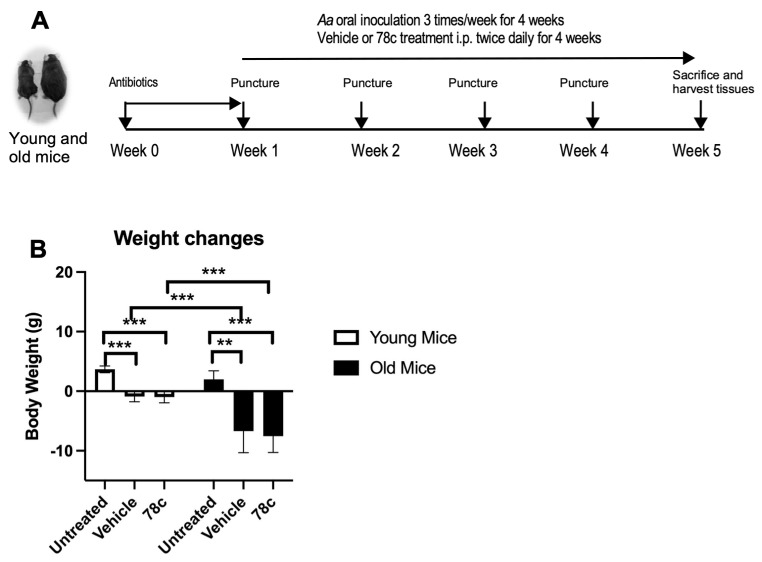
Old C57BL/6J mice lost significantly more body weight during experimental periodontitis than young controls. (**A**) Schematic diagram of mice treatment. Young or old C57BL/6J mice drank antibiotic water (containing 700 μg/mL sulfamethoxazole and 300 μg/mL trimethoprim) for a week to reduce indigenous oral microflora. The experimental periodontitis was initiated by puncturing the palatal gingival tissues with a 27-gauge ½ inch needle (3 punctures on the right and 3 punctures on the left once/week) to allow minor mucosal injury, which was then followed by oral inoculation of the oral pathogen *Aggregatibacter atinomycetemcomitans* (*Aa*) three times/week. Mice were untreated (n = 3), treated with vehicle (n = 10), or treated with 78c (n = 10, 10 mg/kg) by intraperitoneal injection twice daily for 4 weeks. (**B**) Mice’s body weight was measured before and after the 4-week *Aa* inoculation period (** *p* < 0.01, *** *p* < 0.001).

To initiate chronic periodontitis in mice, researchers usually administer an oral pathogen into the oral cavity [28]. However, the C57BL/6 mice were resistant to the conventional oral gavage of an oral pathogen model [29]. Therefore, we modified the conventional oral gavage model by puncturing the palatal gingival tissues with a 27-gauge, ½-inch needle once/week, followed by oral administration of the oral pathogen *Aa* 3 times/week (Figure 1A). This modified procedure causes minor mucosal injury and enhances bacterial colonization in the gingival tissues. To compare NAD^+^ levels in the tissues of old and young mice and determine the therapeutic effects of 78c in mice with experimental periodontitis, old and young mice were untreated (n = 3), treated with vehicle (n = 10), or treated with 78c (n = 10) via intraperitoneal injection twice daily. After 4 weeks of treatment, vehicle-treated young mice lost an average of 0.9 g (3.3%) and 78c-treated young mice lost an average of 1.0 g (3.6%) (Figure 1B). In contrast, vehicle-treated old mice lost an average of 6.7 g (15.9%), and 78c-treated old mice lost an average of 7.5 g (17.2%). There were significant differences in weight loss between the young mice and the old mice groups (n = 10, *** *p* < 0.001). These results supported that old mice displayed poor recovery after periodontal inflammation compared to young controls.

### 2.2. Untreated Old Mice Showed Lower NAD^+^ Levels in the Liver Compared to Young Controls, and Treatment with 78c Elevated NAD^+^ Levels in the Tissues of Both Young and Old Mice

**Figure 2:**
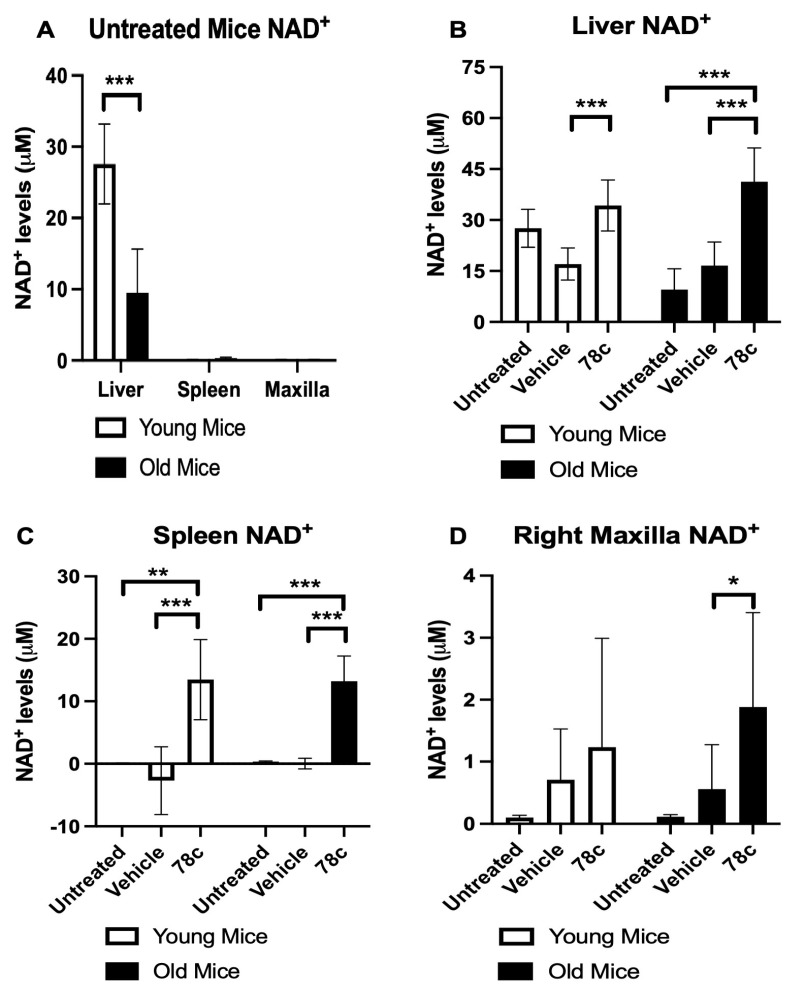
NAD^+^ levels in the liver, spleen, and right maxillary tissues in untreated mice, vehicle-treated mice, and 78c-treated mice. Old or young male C57BL/6J mice were either untreated (n = 3), treated with vehicle and orally administered with the oral pathogen *Aggregatibacter atinomycetemcomitans* (*Aa*) (n = 10), or treated with 78c and orally administered with *Aa* (n = 10) for 4 weeks. (**A**) NAD^+^ levels in the liver, spleen, and right maxillary tissues of untreated mice. (**B**) NAD^+^ levels in the liver of untreated mice, vehicle-treated mice, or 78c-treated mice. (**C**) NAD^+^ levels in the spleen of untreated mice, vehicle-treated mice, or 78c-treated mice. (**D**) NAD^+^ levels in the right maxillary tissues of untreated mice, vehicle-treated mice, or 78c-treated mice. The NAD^+^ levels in the tissues were evaluated by a NAD/NADH assay kit (Sigma Aldrich, St. Louis, MO, USA) (* *p* < 0.05, ** *p* < 0.01, *** *p* < 0.001).

In untreated mice (Figure 2A), old mice displayed a 2.9-fold reduction in NAD^+^ in the liver compared to young controls (n = 3, *** *p* < 0.001). The NAD^+^ levels in the spleen and the right maxilla of untreated mice were too low to be detectable. Treatment with 78c significantly enhanced NAD^+^ levels both in the liver of young mice (2.0-fold) and old mice (2.5-fold) compared with vehicle-treated controls, respectively. Additionally, treatment with 78c significantly increased NAD^+^ levels in the spleens of both young and old mice compared with vehicle-treated controls (n = 10, *** *p* < 0.001). In the right maxillary tissues, 78c-treated old mice significantly increased NAD^+^ by 3.4-fold compared with vehicle-treated controls (n = 10, * *p* < 0.05). We also observed an increase in NAD^+^ in the maxilla tissues of young mice treated with 78c. However, it was not significantly different from vehicle-treated controls.

### 2.3. BMMs Derived from 78c-Treated Old Mice Showed a Significant Decrease in Osteoclastogenesis Compared to Vehicle-Treated Controls

**Figure 3:**
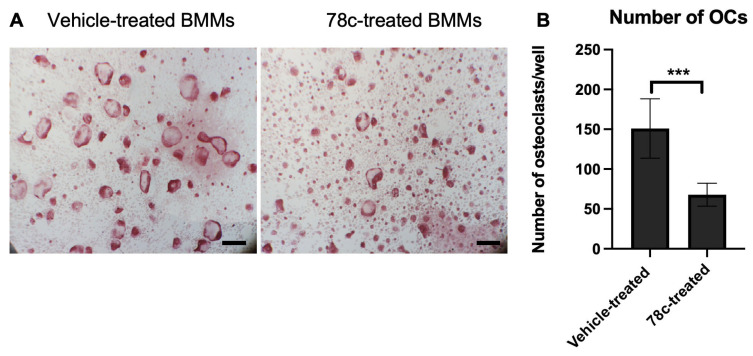
Bone marrow cells derived from 78c-treated old mice reduced RANKL-induced osteoclastogenesis compared with vehicle-treated controls. Bone marrow cells were harvested from vehicle-treated or 78c-treated old mice and cultured in the presence of M-CSF and RANKL for 5 days. (**A**) Representative images of TRAP-stained BMMs derived from vehicle-treated old mice or 78c-treated old mice. Scale bars represent 20 μm. (**B**) Number of TRAP^+^ multinucleated (more than 3 nuclei) osteoclasts (OCs)/well (96-well), (n = 5, *** *p* < 0.001).

To evaluate the effect of 78c in osteoclastogenesis in mice, we harvested bone marrow cells from old mice after 4 weeks of treatment with either 78c or vehicle, and cultured the cells in the presence of 10% L929-conditioned media [27] (containing macrophage colony-stimulating factor, M-CSF) and RANKL (50 ng/mL) for 5 days. In line with our previous study [27], bone marrow cells derived from 78c-treated old mice showed reduced osteoclastogenesis compared with vehicle-treated controls (Figure 3). BMMs derived from 78c-treated old mice showed small osteoclasts and significantly reduced the number of osteoclasts/well 1.8-fold compared with BMMs derived from vehicle-treated controls (n = 5, *** *p* < 0.001).

### 2.4. Treatment with 78c Significantly Reduced Alveolar Bone Loss in Both Young and Old Mice with Experimental Periodontitis Compared with Vehicle Treatment

**Figure 4:**
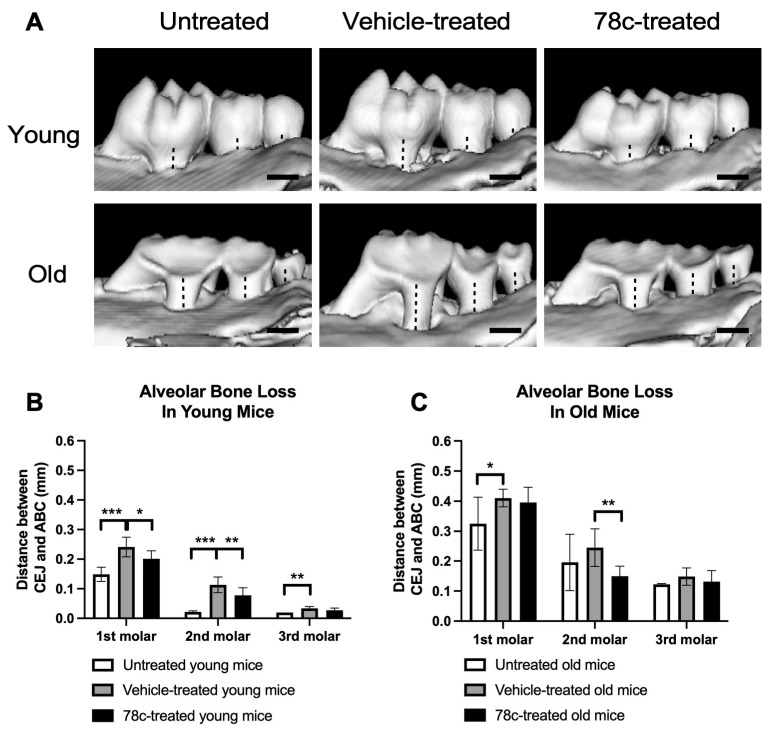
Treatment with 78c alleviated alveolar bone loss in mice with experimental periodontitis. (**A**) Representative micro-CT images of the left maxillary alveolar bone tissues are shown. The scale bars represent 250 μm. Dotted lines illustrate the distances from the cementoenamel junction (CEJ) to the alveolar bone crest (ABC). (**B**) The measured distances between CEJ and ABC in young mice are shown (n = 10, * *p* < 0.05, ** *p* < 0.01, *** *p* < 0.001). (**C**) The measured distances between CEJ and ABC in old mice are shown (n = 10, * *p* < 0.05, ** *p* < 0.01).

To determine the effect of 78c on alveolar bone loss, the left maxillary tissues were scanned by micro-CT. As shown in Figure 4A,B, vehicle-treated young mice significantly increased alveolar bone loss at the 1st, 2nd, and 3rd molars compared with untreated controls. Treatment with 78c significantly reduced alveolar bone loss at the 1st and 2nd molars compared with vehicle treatment. Vehicle-treated old mice also significantly increased alveolar bone loss at the 1st molar, and treatment with 78c significantly attenuated alveolar bone loss at the 2nd molar compared with vehicle treatment. These results support the notion that 78c has a therapeutic effect on alleviating alveolar bone loss in mice.

### 2.5. Old Mice Displayed More Polymorphonuclear Leukocytes (PMN) in the Periodontal Epithelium Compared with Young Controls, and Treatment with 78c Reduced the mRNA Levels of CD38, IL-1β, and TNF in the Gingival Tissues Compared with Vehicle Treatment

**Figure 5:**
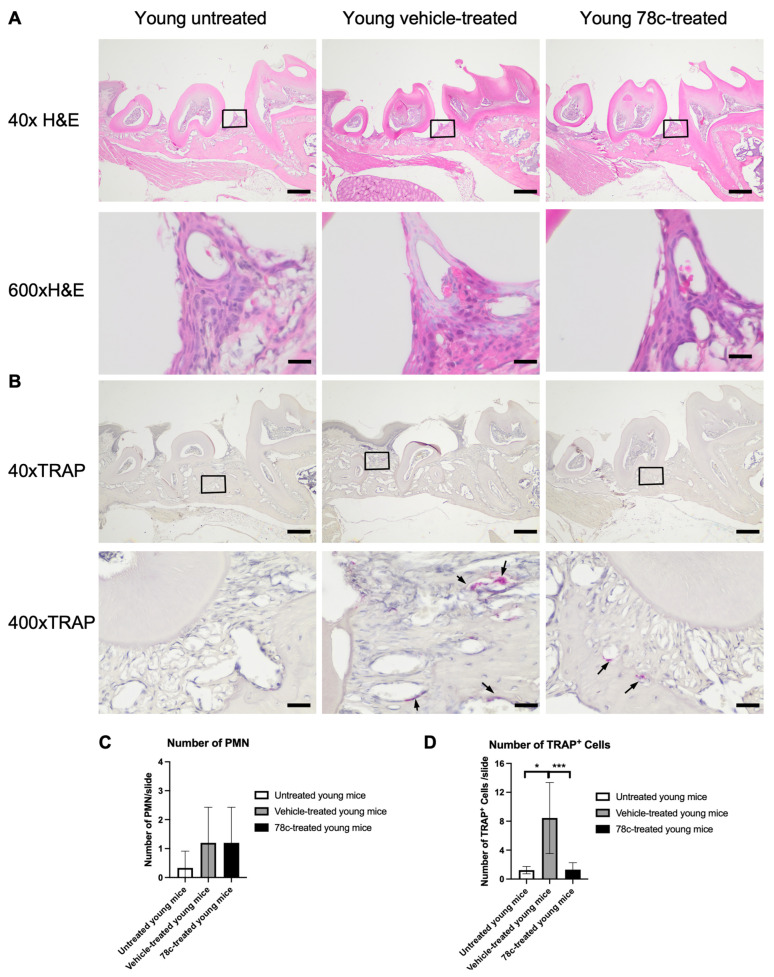
Young mice exhibited no significant inflammation in the periodontal tissues after 4 weeks of treatment. (**A**) Representative images of hematoxylin and eosin (H&E) staining of the maxillary periodontal tissues of untreated young mice, vehicle-treated young mice, and 78c-treated young mice. Images were taken under 40× magnification or 600× magnification. (**B**) Representative images of tartrate-resistant acid phosphatase (TRAP) staining of the maxillary periodontal tissues of untreated young mice, vehicle-treated young mice, and 78c-treated young mice. Images were taken under 40× magnification or 400× magnification. Black arrows indicate TRAP-stained positive cells in the periodontal tissues. The black boxes indicate the magnified region in the periodontal epithelium. The scale bars represent 200 μm in the 40× images, 20 μm in the 400× images, and 30 μm in the 600× images, respectively. (**C**) Number of PMN in the periodontal epithelium per tissue slide. Untreated n = 3, vehicle-treated n = 10, 78c-treated n = 10. (**D**) Number of TRAP^+^ cells/slide. Untreated n = 3, vehicle-treated n = 10, 78c-treated n = 10. (* *p* < 0.05, *** *p* < 0.001).

**Figure 6:**
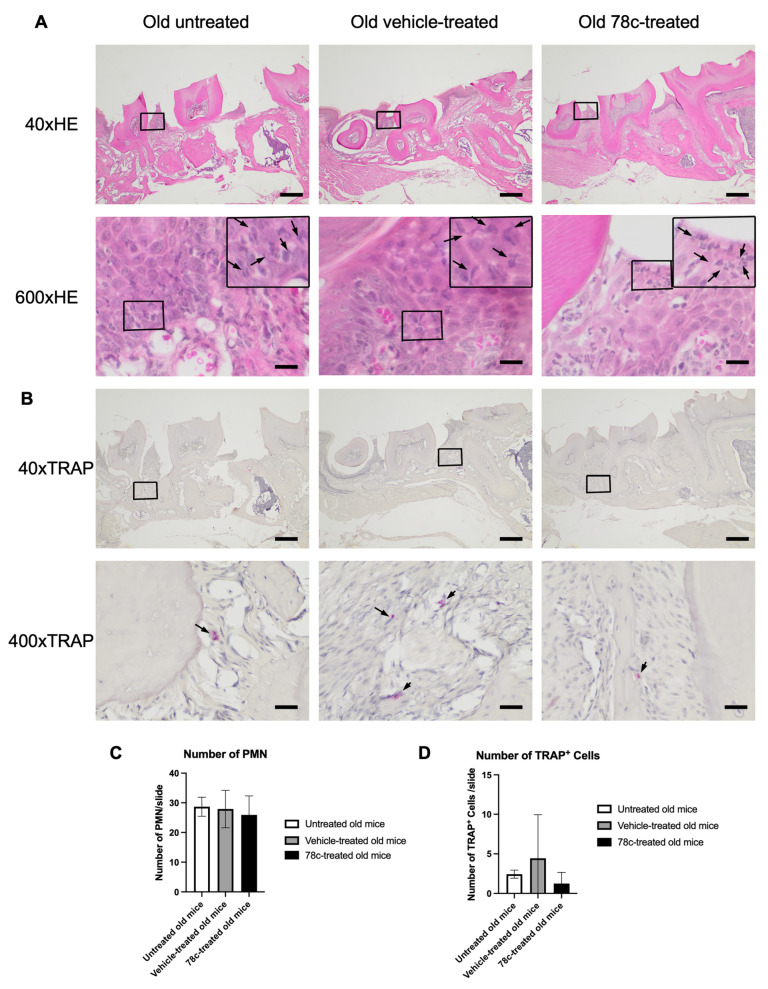
Old mice displayed some polymorphonuclear leukocytes (PMNs) in the periodontal epithelium after 4 weeks of treatment. (**A**) Representative images of hematoxylin and eosin (H&E) staining of the maxillary periodontal tissues of untreated old mice, vehicle-treated old mice, and 78c-treated old mice. Images were taken under 40× magnification or 600× magnification. Black arrows show some PMN in the periodontal epithelium. (**B**) Representative images of tartrate-resistant acid phosphatase (TRAP) staining of the maxillary periodontal tissues of untreated old mice, vehicle-treated old mice, and 78c-treated old mice. Images were taken under 40× magnification or 400× magnification. Black arrows indicate TRAP-stained positive cells in the periodontal tissues. The black boxes indicate the magnified region in the periodontal epithelium. The scale bars represent 200 μm in the 40× images, 20 μm in the 400× images, and 30 μm in the 600× images, respectively. (**C**) Number of PMN in the periodontal epithelium per tissue slide. Untreated n = 3, vehicle-treated n = 10, 78c-treated n = 10. (**D**) Number of TRAP^+^ cells/slide. Untreated n = 3, vehicle-treated n = 10, 78c-treated n = 10.

To determine whether treatment with 78c could alleviate periodontal inflammation compared with vehicle treatment, the left maxillary tissues underwent histological processing and sectioning. Hematoxylin and eosin (H&E) staining of periodontal tissues of young mice (Figure 5) did not show any significant inflammation in the periodontal tissues. In contrast, H&E staining of periodontal tissues of old mice (Figure 6) displayed some PMN in the epithelium in untreated mice, vehicle-treated mice, and 78c−treated mice. The mRNA levels of CD38, IL-1β, IL-6, and TNF were undetectable in the right maxillary tissues of both young and old mice after 4 weeks of treatment. These results support the idea that old mice have an increased inflammatory response compared with young mice, and that periodontal inflammation mostly resolved after 4 weeks of treatment. Although we observed a few small TRAP-stained positive cells in the periodontal tissues of both young and old mice, they are mainly single-nucleus pre-osteoclasts, not multinucleated osteoclasts. Treatment with 78c significantly reduced the number of TRAP^+^ cell in the periodontal tissues of young mice (Figure 5D).

**Figure 7:**
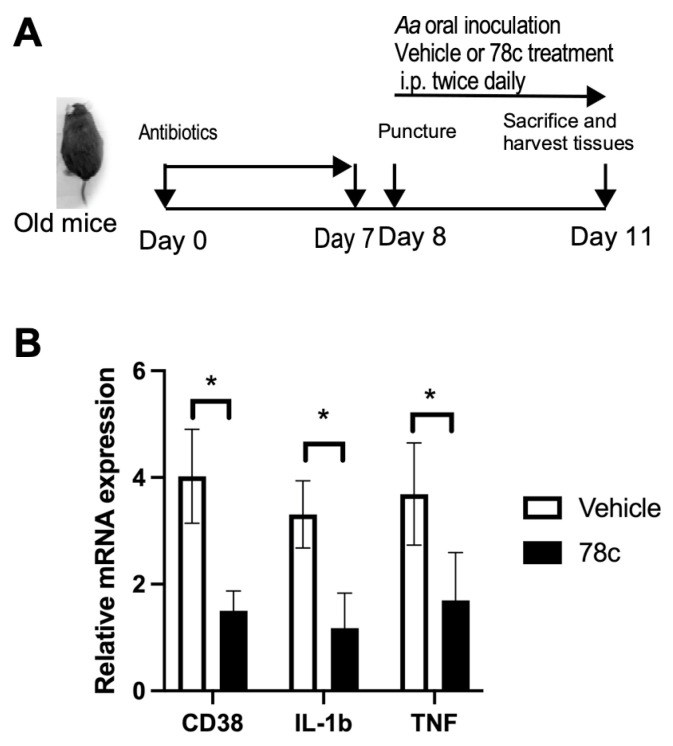
Treatment with 78c in old C57BL/6J mice significantly reduced the mRNA levels of CD38, IL-1β, and TNF in the gingival tissues compared with vehicle treatment. (**A**) Schematic diagram of mice treatment. Old C57BL/6J mice drank antibiotic water (containing 700 μg/mL sulfamethoxazole and 300 μg/mL trimethoprim) for a week to reduce indigenous oral microflora, then drank normal water for 1 day. The palatal gingival tissues were punctured by a 27-gauge ½ inch needle (3 punctures on the right and 3 punctures on the left), followed by oral inoculation of the oral pathogen *Aggregatibacter atinomycetemcomitans* (*Aa*). Mice were untreated (n = 3), treated with vehicle (n = 3), or treated with 78c (n = 3) by intraperitoneal injection twice daily for 3 days. (**B**) The relative mRNA levels of CD38, IL-1β, and TNF in the left gingival tissues 3 days after initiation of experimental periodontitis were evaluated by RT-PCR (* *p* < 0.05).

To determine the effect of 78c on CD38 and pro-inflammatory cytokine levels in the periodontal tissues, we performed another short-term study (Figure 7A). Old mice were punctured once with a 27-gauge needle, then administered *Aa* orally every other day for 3 days. Mice were treated with either vehicle or 78c twice daily by intraperitoneal injection. The left gingival tissues were harvested on day 3. As shown in Figure 7B, treatment with 78c significantly reduced the mRNA levels of CD38, IL-1β, and TNF in the left gingival tissues compared with vehicle-treated controls. The IL-6 mRNA levels were undetectable in the gingival tissues. These data support that treatment with 78c inhibited CD38 and suppressed IL-1β and TNF levels in old mice with experimental periodontitis.

## 3. Discussion

In this study, treatment with the CD38-specific inhibitor (78c) significantly increased NAD^+^ levels in the livers and spleens of both young and old mice, consistent with a previous study [30] showing that 78c treatment in old mice increased NAD^+^ in tissues. We observed a large variation in NAD^+^ levels in the maxillary tissues compared with those in the liver or spleen. This might be caused by differences in the components of the maxillary tissues (including bone and muscles) compared with those in the liver and spleen. Because different tissues have different NAD^+^ contents, measuring NAD^+^ levels in 20 mg of mixed bone and muscle tissue can lead to large variation. We observed a significant weight loss in old mice with experimental periodontitis compared to young controls (Figure 1). We also observed that the old mice ate less soft food compared with young controls. Therefore, the weight loss in the old mice might be associated with less food intake under stress condition during this study.

In this study, we observed an increase in CD38, IL-1β, and TNF mRNA levels in gingival tissues 3 days after the first puncture, followed by *Aa* inoculation in the oral cavity in mice (Figure 7). However, the mRNA of CD38, IL-1β, and TNF was not detectable at the end of 4 weeks in the maxillary tissues (7 days after the last puncturing followed by *Aa* inoculation in the oral cavity in mice). These results suggest that the inflammation was induced in the early period following needle puncturing and *Aa* inoculation and that the inflammation resolved 7 days after puncturing. The alveolar bone loss we observed at the end of 4 weeks (Figure 4) was the result of repeated increases in inflammatory cytokines in the periodontal tissues following puncturing and *Aa* inoculation, which led to osteoclastogenesis and alveolar bone loss. Our previous study [27] showed that 78c suppressed RANKL-induced osteoclastogenesis and bone resorption, and reduced pro-inflammatory cytokine expression in murine BMMs infected with *Aa*. The mechanisms associated with 78c inhibiting osteoclastogenesis were 78c decreased podosome (basic cell adhesion unit) components, such as PI3K, Pyk2, Src, F-actin, integrins, paxillin, and talin [27]. Consequently, treating mice with 78c may have reduced alveolar bone loss primarily by inhibiting RANKL-induced cell adhesion and fusion, thereby preventing the formation of multinucleated osteoclasts. The mechanisms associated with 78c suppressing inflammatory cytokines were 78c suppressed PI3K, NF-κB, and MAPKs induced by oral pathogens (*Aa* or *Pg*) [27]. In this study, we observed increased PMN infiltration in the periodontal epithelium of untreated old mice (Figure 6) and greater alveolar bone loss in these mice compared to young controls (Figure 4). This is likely related to enhanced age-related inflammation known as inflammaging [13,14].

Although 78c is a CD38-specific inhibitor, it exhibited some off-target effects, including inhibition of IL-1β, IL-6, and TNF expression induced by oral pathogens and suppression of RANKL-induced osteoclastogenesis. Our previous study [27] showed that knockdown of CD38 by a CD38 shRNA in murine BMMs enhanced NAD^+^ levels in the presence or absence of bacterial infection. However, the CD38 shRNA treatment showed limited effects on IL-1β, IL-6, and TNF levels induced by *Aa* or *Pg* compared with the control shRNA treatment. Additionally, treatment with the CD38 shRNA enhanced RANKL-induced osteoclastogenesis compared with treatment with the control shRNA [27]. These results suggest that the increase in NAD^+^ might not directly regulate cytokine production or RANKL-induced osteoclastogenesis.

Rodents have been used to investigate a variety of mechanisms underlying oral diseases because the periodontal anatomy of rodent molars is similar to that of humans [3]. Common techniques used in rodent models of periodontal disease include placement of ligatures in the gingival sulcus, oral gavage with periodontal pathogens, and oral administration of dextran sulfate sodium (DSS) to induce damage to the gastrointestinal tissues, which can subsequently result in oral mucosal inflammation and alveolar bone loss [3]. The ligature placement causes severe mucosal damage and bacterial colonization, leading to acute periodontal inflammation. Previously, we placed ligatures around the 2nd molars of 2-month-old C56BL/6J mice for 15 days, resulting in severe alveolar bone loss [31]. Oral administration of DSS to 11–12-week-old BALB/c mice caused colitis and diarrhea [32]. Because the old mice do not tolerate ligature placement or DSS administration, the majority of age-related periodontitis studies have adopted an oral inoculation approach using an oral pathogen [33]. However, previous studies showed that C57BL/6 mice were resistant to conventional oral gavage of an oral pathogen model [29]. We modified the approach by puncturing the palatal gingival tissues with a needle, followed by oral inoculation with the oral pathogen *Aa*. This technique induced a mild inflammation, and the mice resolved it after 4 weeks of treatment. We did not observe a significant difference in H&E or TRAP staining between young and old mice treated with 78c or vehicle (Figure 5 and Figure 6). Future studies are required to modify the existing experimental periodontitis approach to detect differences in the inflammatory response and TRAP-stained osteoclasts between the 78c-treated and vehicle-treated groups.

This study also had some other limitations. First, *Aa* infection is associated with 90% of localized aggressive periodontitis in juveniles [34] and is not a major oral pathogen in aging-associated periodontitis [35]. Because *Aa* is a facultative anaerobic organism that can grow under conditions with or without the presence of oxygen, *Aa* can survive in the oral cavity of mice. In contrast, the oral pathogen *Pg* requires strictly anaerobic conditions and dies in the oral cavity upon exposure to oxygen. In the future, it is necessary to determine whether treatment with 78c in mice can alleviate inflammatory bone loss in mice infected with other oral pathogens associated with aging-associated periodontitis, including *Porphyromonas gingivalis* (*Pg*), *Tannerella forsythia*, or *Treponema denticola* [35]. Second, we used only 3 mice/group to determine CD38, IL-1β, and TNF mRNA levels in gingival tissues. Future studies should use a large cohort of mice to determine whether treatment with 78c reduces CD38, IL-1β, and TNF protein levels in gingival tissues. Third, this study did not determine the effect of 78c on host-microbe interaction in mice. Previous studies [36] have demonstrated that age-related diseases and various medications contribute to microbial dysbiosis, and that oral dysbiosis can affect the gut microbiome, creating a cascade that impacts the gut–brain axis. Future studies are required to determine the effect of 78c on microbes in the oral cavity or gut.

Currently, the gold-standard treatments of periodontitis include scaling and root surface debridement to remove bacterial plaque. There are still patients or sites that show poor response to these treatments. This could be due to sustained dysbiosis, bacterial invasion of periodontal tissues, or a non-resolving chronic inflammatory response. In this study, we demonstrated that treatment with the CD38-specific inhibitor (78c) reduced the mRNA levels of CD38, IL-1β, and TNF in gingival tissues, increased NAD^+^ levels in the liver and spleen, and alleviated alveolar bone loss in mice with experimental periodontitis. Additionally, previous studies also showed that treatment with 78c increased the lifespan and healthspan of naturally aged mice [37] and improved several physiological and metabolic parameters of aging, including glucose tolerance, muscle function, exercise capacity, and cardiac function in mouse models of natural and accelerated aging [30,37]. Our and other studies’ results support the notion that treatment with 78c can serve as a promising therapeutic strategy for periodontitis, alleviating periodontal inflammatory bone loss, enhancing NAD^+^_,_ and subsequently promoting the lifespan and healthspan of human beings.

## 4. Materials and Methods

### 4.1. Animals and Reagents

Old (18-month-old) and young (2-month-old) male C57BL/6J mice were sourced from Jackson Laboratory (Bar Harbor, ME, USA). They were kept under a 12-h light/12-h dark cycle under specific pathogen-free conditions with free access to food and water. All procedures involving animals were approved by the Institutional Animal Care and Use Committee at the Medical University of South Carolina (IACUC-2021-01287). 78c was purchased from Cayman Chemical (Ann Arbor, MI, USA). Dimethyl sulfoxide (DMSO), PEG400, and (2-hydroxypropyl)-γ-cyclodextrin were purchased from Fisher Scientific (Pittsburgh, PA, USA). 78c was first dissolved in DMSO (100 mM), followed by dilution in 15% PEG400 and 80% of 15% (2-hydroxypropyl)-γ-cyclodextrin in citrate buffer (pH 6.0) with a final 5% DMSO [30]. The vehicle consisted of 5% DMSO, 15% PEG400, and 80% of 15% (2-hydroxypropyl)-γ-cyclodextrin in citrate buffer (pH 6.0) [30]. 78c or vehicle was sterilized by filtering through a sterile bottle-top filter with a 0.22 μm membrane (Fisher Scientific), aliquoted, and stored at −20 °C.

### 4.2. Bacterial Culture

The oral bacterial pathogen *Aggregatibacter actinomycetemcomitans* (*Aa*, ATCC 43718) was obtained from the American Type Culture Collection. *Aa* was streaked in brain-heart infusion agar (Fisher Scientific) and cultured in brain-heart infusion broth (Fisher Scientific) at 37 °C with 10% CO_2_. The *Aa* culture media was centrifuged, and the *Aa* cell pellets were washed with PBS and resuspended in PBS containing 1.5% carboxymethylcellulose (Fisher Scientific). The bacterial concentration was adjusted to 1 × 10^10^ colony forming units (CFU)/mL by measuring bacterial optical density at 600 nm of 10× diluted of bacteria (OD_600_ = 1 was equal to 1 × 10^9^ CFU/mL of *Aa*).

### 4.3. Animal Treatment

Mice drank antibiotic water (containing sulfamethoxazole 700 µg/mL and trimethoprim 300 µg/mL, Fisher Scientific) for 7 days to reduce indigenous oral microflora, then drank normal water for 1 day to remove residual antibiotics. To initiate experimental periodontitis, the mice were first sedated by inhaling isoflurane. Then, the palatal gingival tissues of mice were punctured (3 punctures on the left and 3 punctures on the right) by a 27 gauge ½ inch needle (BD Diagnostic System, Franklin Lakes, NJ, USA) once per week to cause minor mucosal damage followed by oral inoculation of the oral pathogen *Aa* (5 × 10^8^ colony forming units, 50 μL) via a wide pore pipette tip (3 times/week for indicated days in figures). This procedure allows *Aa* to easily attach to the gingival mucosa and subsequently cause periodontal inflammation. The mice were either untreated, treated with vehicle, or treated with 78c (10 mg/kg) by intraperitoneal injection twice daily for the indicated days, as shown in the figures. Soft foods were provided for the mice after *Aa* administration. After euthanizing the mice, the liver, spleen, bone marrow, maxilla, or gingival mucosa were harvested.

### 4.4. NAD^+^ Assay

The liver, spleen, and right maxillary tissues were stored at −80 °C. The tissues were homogenized using a mortar and pestle. NAD^+^ levels were determined using a NAD^+^/NADH assay kit (Sigma-Aldrich, St. Louis, MO, USA) in 20 mg of tissue, according to the manufacturer’s instructions.

### 4.5. Osteoclastogenesis Assay

Murine bone marrow cells were collected from five old mice treated with a vehicle and five old mice treated with 78c after 4 weeks of treatment. Bone marrow was flushed from the tibia and femur using complete minimal essential media (MEM)-α (Fisher Scientific), supplemented with 10% FBS, 100 U/mL penicillin, and 100 µg/mL streptomycin. Bone marrow cells were cultured in complete MEM-α media supplemented with 20% L929 conditioned media (containing macrophage colony-stimulating factor, M-CSF) [27] for 2 days to allow bone marrow stromal cells to attach onto the plates. The suspended bone marrow cells were transferred to new Petri dishes and cultured in complete MEM-α medium supplemented with 20% L929-conditioned medium until they attached. The attached bone marrow-derived monocytes (BMMs) were plated in a 96-well plate and cultured in complete MEM-α medium supplemented with 10% conditioned L929 medium and recombinant human RANKL (50 ng/mL, PeproTech, Cranbury, NJ, USA). The media was refreshed every two days. After five days of RANKL treatment, osteoclasts were stained with Tartrate-Resistant Acid Phosphatase (TRAP) using a leukocyte acid phosphatase kit (Millipore Sigma, Burlington, MA, USA).

### 4.6. Micro-Computed Tomography (Micro-CT) Scanning and Alveolar Bone Loss Assessment

The left maxillary tissues were fixed with 10% formalin for 2 days and then stored in 70% ethanol at 4 °C. They were scanned using a cone-beam µ-CT40 system (Scanco Medical AG, Brüttisellen, Switzerland). The resulting three-dimensional micro-CT images were visualized using the GE Healthcare MicroView software version 2.1.0.0. Alveolar bone loss was accessed by measuring the distance from the cementoenamel junction (CEJ) to the alveolar bone crest (ABC) using Adobe Photoshop CS5.1.

### 4.7. Tissue Processing, Staining, and Evaluation

After 2 days of 10% formalin treatment and storage in 70% ethanol, the left maxillary tissues were decalcified in 20% EDTA for 4 weeks. Subsequently, they were processed and embedded in paraffin. Three consecutive 5 µm sagittal sections were cut every 100 μM from a fixed position (starting from the point at which the roots of the second molar become clearly visible). These sections were stained with hematoxylin and eosin (H&E) for general histology or with tartrate-resistant acid phosphatase (TRAP) to evaluate osteoclasts. Images were captured using an Olympus BX43 microscope (Olympus Corporation of the Americas, Center Valley, PA, USA). The mean number of PMNs, or the number of TRAP^+^ cells per tissue slide, was defined as the value for one animal.

### 4.8. RNA Extraction and Real-Time PCR

Total RNA was extracted from maxilla or gingival tissues using TRIZOL reagent (ThermoFisher Scientific, Waltham, MA, USA). Complementary DNA (cDNA) was then synthesized from 1 μg of total RNA with a TaqMan reverse transcription kit (Life Technologies, Carlsbad, CA, USA). Real-time PCR analysis was conducted on a StepOnePlus Real-Time PCR System (Life Technologies) under the following conditions: 50 °C for 2 min, 95 °C for 10 min, and 40 cycles of 95 °C for 15 s, and 60 °C for 1 min. Primers for the target genes: CD38 (Mm00483143_m1), IL-1β (Mm00434228_m1), IL-6 (Mm00446190_m1), TNF (Mm00443258_m1), and β-actin (Mm02619580_g1) were sourced from Life Technologies. Amplicon concentrations were calculated by comparing threshold cycle values to standard curves for each primer. Sample mRNA levels were normalized against β-actin and expressed as fold changes relative to control groups.

### 4.9. Statistical Analysis

The data were analyzed with a one-way ANOVA, using Dunnett’s or Tukey’s multiple comparison tests for comparisons involving more than three groups. For comparison between two groups, the unpaired Student’s t-test with Welch’s correction was used. All statistical analyses were conducted using GraphPad Prism software (Version 10.6.1, GraphPad Software Inc., La Jolla, CA, USA). Results are presented as means ± standard error of the means (SEM) across multiple independent experiments. A *p*-value of 0.05 or less was deemed statistically significant.

## Data Availability

The raw data supporting the conclusions of this article will be made available by the authors on request.
